# Potential roles of psychological and oxidative stress in insulin resistance: a cohort-based study

**DOI:** 10.1186/s13098-020-00566-8

**Published:** 2020-07-08

**Authors:** Miroslaw Janczura, Jerzy Dropinski, Anna Gielicz, Katarzyna Kotula-Horowitz, Teresa Iwaniec, Andrzej Stanisz, Rafal Rosa, Teresa B. Domagala

**Affiliations:** 1 Faculty of Health Sciences, Jagiellonian University School of Medicine, Krakow, Poland; 2 Department of Internal Medicine, Jagiellonian University School of Medicine, Krakow, Poland; 3 Department of Internal Medicine, Health Care Centre of the Ministry of the Interior and Administration, Krakow, Poland; 4 Department of Bioinformatics and Telemedicine, Jagiellonian University School of Medicine, Krakow, Poland; 5 Department of Anesthesiology and Intensive Care, Health Care Centre of the Ministry of the Interior and Administration, Krakow, Poland; 6 Department of Medical Biochemistry, Jagiellonian University School of Medicine, 31-034 Krakow, Poland

**Keywords:** Insulin resistance, Psychological stress, Cortisol, Oxidative stress

## Abstract

**Background:**

The present study investigated the relationships between psychological stress indices and oxidative stress marker, also when combined with emergent insulin resistance (IR), in the non-diabetic, middle-aged subjects, exposed to frequent/chronic psychological stressors.

**Methods:**

Cross-sectional data from a cohort of non-diabetic police officers (n = 234; 19F), aged 27–56 years, were used. Plasma inflammatory (CRP, TNF-α), oxidative stress (free 8-iso-prostaglandin F_2α_; 8-iso-PGF_2α_) markers, and insulin were measured. The value of homeostasis model assessment of IR index (HOMA-IR) was assumed the threshold value of IR, i.e. 2.04. Free cortisol in urine and perceived stress (psychological stress indices) were also measured.

**Results:**

In the IR subjects, most biochemical variables, inflammatory markers and urine cortisol were significantly higher, as compared to the non-IR ones. Psychological stress indices were associated with plasma 8-iso-PGF_2α_ [B = 0.139, 95% CI (0.048, 0.230), p = 0.002, and B = 0.007, 95% CI (0.0006, 0.014), p = 0.03; for perceived stress level and cortisol, respectively]. Positive associations were established between plasma 8-iso-PGF_2α_ [B = 0.069, 95% CI (0.016–0.120), p = 0.01] and urine cortisol [B = 0.003, 95% CI (0.0003, 0.005), p = 0.02] with HOMA-IR. Metabolic syndrome, as defined by IDF criteria, was established in 110 study subjects, whereas 136 of them were hypertensive. Waist circumference [B = 0.056, 95% CI (0.039, 0.074), p < 0.0001], and systolic blood pressure [B = 0.009, 95% CI (0.00003, 0.018), p = 0.04] were positively associated with HOMA-IR, whereas the association of HDL cholesterol [B = − 0.597, 95% CI (− 1.139, − 0.055), p = 0.03] was a negative one. Cortisol [OR = 1.007, 95% CI (1.002, 1.012), p = 0.006], and 8-iso-PGF_2α_ [OR = 1.103, 95% CI (1.010, 1.201), p = 0.02] affected the incidence of IR. After adjustment for metabolic syndrome (or its components), age, sex, and current smoking, the effects became non-significant. Out of metabolic syndrome components, waist circumference [OR 4.966, 95% CI (2.29, 10.751), p = 0.00004] and hypertriglyceridemia [OR 1.993, 95% CI (1.063, 3.736), p = 0.03] increased the chance of IR incidence.

**Conclusions:**

Both psychological stress indices were associated with oxidative stress, but only cortisol with HOMA-IR. In the subjects exposed to frequent/chronic psychological stressors, cortisol and oxidative stress marker affected IR incidence, being statistically attenuated, though, following adjustment for metabolic syndrome, or its components.

## Background

Insulin resistance (IR) is believed to be crucially instrumental in the development of metabolic syndrome (MetS), as well as may indirectly promote cardiovascular disease (CVD) through the development of abnormal glucose and lipid metabolism, hypertension, and pro-inflammatory status [1]. MetS affects up to 30% of the population in the developed countries, while central obesity makes up one of its components, being also a key contributor to insulin resistance (IR), type 2 diabetes, and increases overall risk of CVD [1, 2].

Psychological stress is recognised as an important risk factor status in CVD, diabetes, and MetS [3–5]. The Perceived Stress Scale (PSS) is one of the more popular, subjective tools for assessing psychological stress, even though only few studies have addressed the association between perceived stress and diabetes [4, 6, 7]. In response to physiological or psychological stressors, resulting in the secretion of cortisol from the adrenal gland, a cascade of physiological events follows. Hypercortisolism may induce IR, and lead to type 2 diabetes by inhibiting insulin secretion, as well as promote development of visceral adiposity, while activating lipolysis and free fatty acid release [8]. Chronic stress may also impair the feedback mechanisms which return these hormonal systems to normal, resulting in chronic elevation in cortisol levels, though inter-individual differences in the hypothalamic–pituitary–adrenal (HPA) axis function may be causally linked to higher hazard of succumbing to certain stress-related metabolic disorders [3, 9]. The changes in the normal level of cortisol secretion, may account for an accelerated metabolic rate and subsequent generation of reactive oxygen species (ROS), having also been associated with a higher level of oxidative stress markers [10, 11]. Oxidative stress is deemed of great significance in the aetiology origin of many diseases, in view of the association of the end products of free radicals-mediated oxidative stress with obesity, IR, dyslipidemia, and hypertension [10, 12, 13]. F2-isoprostanes, prostaglandin-like molecules generated in vivo are the most reliable markers of lipid oxidation and its measurement, widely used to assess oxidative stress in various diseases. Out of all oxidized fatty acids, the 8-iso-prostaglandin F_2α_ (8-iso-PGF_2α_), also referred to as 15-F2t-isoprostane (or iPF2α-III), has been the most studied isomer, also known as an in vivo biomarker of oxidative stress [14]. Published data on oxidative stress in humans in relation to IR remain scarce and inconclusive. Some cross-sectional studies had demonstrated in humans a positive association between urine 8-iso-PGF_2α_ and IR, even though no such relationships were corroborated by other studies [15–17].

In our previous study, we managed to demonstrate that perceived stress was associated with coronary heart disease (CHD) and MetS [5]. There are no published studies, though, taking into account both the psychological and oxidative stress, and their relations with IR. As police officers are widely acknowledged to be exposed to more stressors than individuals pursuing other occupations, we set out to assess the associations between the indices of psychological stress (self-perception, cortisol level), with oxidative stress, and IR. In line with our working hypothesis, psychological stress is believed to be associated with increased systemic oxidative stress and incidence of IR.

## Methods

### Study population

We carried out a cross-sectional study on the cohort of police officers from the southern region of Poland, who had volunteered for CHD screening at the University Hospital in Krakow. The present cohort study is focused on the police officers, as the individuals particularly exposed to a highly stressful occupation. The following inclusion criteria were adopted: active work as a police officer for a minimum period of 5 years (the median duration of work was 15 years (range 6–27), whereas the following ones prompted exclusion from the study protocol: diabetes mellitus, history of myocardial infarction or stroke, cancer, congestive heart failure, atrial fibrillation, current anticoagulant therapy, liver injury, or kidney insufficiency. Subjects with arterial hypertension and lipid disorders (defined as the ones previously diagnosed and treated with statins, or had serum total cholesterol > 5.2 mmol/L) were considered eligible. The subjects who had quit smoking at least 6 months previously were included as non-smokers. As established through the interviews individually conducted with each study subject by the investigators, none of the subjects had been following any alternative dietary profile and/or used any food supplementation with his standard dietary intake. Out of the 240 subjects who underwent medical examinations, ultimately 234 (19F), aged 27–56 years, complied with the inclusion criteria.

Physical examination, medical and structured interviews were applied to collect personal and clinical information. Anthropometric measurements, i.e. circumference of waist and hips, height, and body weight, were collected. Obesity was defined as BMI > 30.0 kg/m^2^, and overweight as BMI > 25.0 kg/m^2^. Atherogenic dyslipidemia was construed as: triglycerides ≥ 1.7 mmol/L and HDL-cholesterol < 1.03/1.29 mmol/L (men/women). MetS was defined in line with the criteria proposed by the International Diabetes Federation (IDF), i.e. waist circumference ≥ 94/80 cm (men/women), fasting glucose ≥ 5.6 mmol/L, triglycerides ≥ 1.7 mmol/L, or specific treatment for this lipid abnormality, HDL-cholesterol < 1.03 mmol/L in men, and < 1.29 mmol/L in women, systolic/diastolic blood pressure ≥ 130/≥ 85 mmHg, or treatment of previously diagnosed hypertension [18].

### Clinical tests

Comprehensive results of specific clinical tests, e.g. computed tomography coronary angiography, exercise electrocardiography, had previously been addressed [5]. Imaging studies, aimed at measuring the surrogate markers of atherosclerosis [flow-mediated dilation [FMD] of brachial artery, and intima-media thickness (IMT)] were also completed using an ultrasonograph (Sequoia 512, Mountain View, Ca, USA) with a 6 MHz linear transducer.

### Measurement of self-perceived stress

Perceived stress was measured with the aid of Cohen’s 10-item Perceived Stress Scale (PSS-10) in Polish adaptation [6]. Specifically structured questions were designed to probe the respondents on how unpredictable, uncontrollable, and overloaded they actually found their lives. Participants were asked to respond to each question on a 5-point Likert scale ranging from 0 (never) to 4 (very often), indicating how often they have felt or thought in a certain way within the past month. Scale scores ranged 0-40, with the higher scores indicating higher levels of stress. The reliability of the scale was assessed by Cronbach’s alpha, while exploratory and confirmatory factor analyses were applied to evaluate validity of PSS-10. The PSS-10 demonstrated good reliability, as Cronbach’s alpha was 0.85 (0.84–0.86).

### Biochemical measurements

Blood sampling was taken between 8:00 a.m. and 8:30 a.m. (after a 12-h overnight fast). Blood samples were collected into the tubes (Becton–Dickinson, Franklin Lakes, NJ, USA) with a clot activator, containing 3.2% sodium citrate (final blood-to-anticoagulant ratio 9:1), and into the ones with EDTA (final concentration 1.8 mg/mL). Following a 10-min long centrifugation at 3500 rpm, plasma and serum got separated within 1 h.

A 24 h urine samples collection proved a non-feasible approach, as the police officers worked shifts. Consequently, the subjects were instructed to pass their urine (midstream) immediately upon waking, and then have another urine sample collected 1 h afterwards. Besides, our study population consisted of active-duty policemen, working in a shift work system, which rendered a 24-h urine collection non-feasible. As plasma cortisol concentration is at its highest 30–45 min after waking up, we measured cortisol in the urine samples obtained from the second emptying of the bladder (1 h after waking up). All urine samples were collected at home, while in the laboratory they were centrifuged for 10 min at 3500 rpm. The samples of urine, separated plasma, and serum were stored at − 80 °C for 3–4 months, pending assays.

Total cholesterol and triglycerides were determined by an enzymatic method, while LDL cholesterol was calculated with the Friedewald formula. Basic biochemical tests (liver enzymes, glucose, urea, uric acid and creatinine (serum, urine) were carried out with the aid of Vitros 350 Chemistry System (Ortho Clinical Diagnostics). Fibrinogen level in plasma was determined by coagulometry using the BCT camera (Behring Coagulation Timer, Dade-Behring, Germany) and the Multifibren U kit. Ultrasensitive CRP (hs-CRP) in the serum was determined by nephelometry (BN II System, Siemens), using CardioPhase assay (Siemens Healthcare Diagnostics Products GmbH, Marburg, Germany). Tissue necrotic factor-α (TNF-α) in the plasma was measured by ELISA (Quantikine HS Human TNF-α, R&D Systems, England). Cortisol level in the urine samples was determined by a commercially available enzyme-linked immunosorbent assay (ELISA Cortisol Assay, R&D Systems, England) with absorbance detection (BioTek Synergy HTX Multi-Mode Microplate Reader, USA). Urinary content of cortisol was expressed as ng/mg of creatinine. The intra- and inter-assay coefficients of variation were 6.3% and 8.3%, respectively.

Insulin in the serum was determined by an immunoassay using a commercial kit (Insulin ELISA, DRG Instruments GmbH; Germany). HOMA-IR index was calculated as fasting insulin x fasting glucose/22.5. Value of HOMA-IR > 80‰ designated in the subgroup of police officers (n = 33) without any metabolic abnormalities was assumed the threshold of IR, i.e. 2.04. The intra- and inter-assay coefficients of variation were 3.7% and 5.3%, respectively.

Plasma free isoprostane 8-iso-PGF_2α_ was determined by gas chromatography coupled with mass spectrometry (GC–MS) [19]. In order to prevent artefactual oxidation ex vivo, 25 µl of 1% butylated hydroxytoluene (BHT) was added to 1 ml of plasma. The measurements were carried out using a mass spectrometer (MS Engine 5989B Hewlet Packard, Palo Alta, CA, USA) connected to a gas chromatograph (GC 5890 series II Hewlet Packard, Palo Alta, CA, USA). The intra- and inter-assay coefficients of variation were 3.35% and 5.82%, respectively.

### Statistical analysis

The normality of data distribution was verified using the Shapiro–Wilk’s test. Continuous variables are presented as median and 95% confidence interval (CI). For comparison of the variables between groups, due to lack of normality, the Mann–Whitney U test was applied. Categorical data were expressed as frequencies (percentages), and their differences analysed using the Chi square test. In the regression models, glucose was not considered, as it was included in the HOMA-IR formula. Univariate and multivariate regression models were applied to assess the relationships between the indices of psychological stress with oxidative stress marker, and HOMA-IR. The associations with HOMA-IR were also assessed in the regression model comprising urine cortisol, plasma 8-iso-PGF_2α_, and the variables whose values, when exceeding the reference range (i.e. abnormal), are deemed the key identification attributes of MetS (waist circumference, triglycerides, HDL cholesterol, systolic blood pressure).

To assess the impact of variables (cortisol, 8-iso-PGF_2α_, atherogenic dyslipidemia, MetS and its components) on the incidence of IR, logistic regression models were applied. In the logistic regression models, cortisol and 8-iso-PGF_2α_ were used as the continuous, while atherogenic dyslipidemia, MetS, and its components were used as the dichotomous, independent variables. The resulting odds ratios (ORs) and 95% confidence intervals (CIs) were reported. The multivariate regression models were adjusted for age, sex, and current smoking. In multivariate models, multicollinearity was not evidenced. For evaluating the correlations between respective variables, Spearman’s rank correlation coefficient was applied. P < 0.05 indicated statistical significance. Statistical analysis was pursued with STATISTICA 13.1 PL.

## Results

Clinical characteristics of the study subjects stratified by IR status are presented in Table 1. The IR subjects, as compared to the non-IR ones, showed higher (although not significantly; p = 0.06) prevalence of stable CHD, diagnosed through typical clinical coronary symptoms and positive exercise ECG testing (Table 1). Coronary artery plaques were found in 32 (33.33%) in the IR, and 13 (21.31%) in the non-IR subjects (p = n.s.). Only carotid IMT value was significantly higher in the IR group (p = 0.001), as evidenced by the ultrasound imaging measurements. The respective groups (IR vs. non-IR) differed in FRS (p = 0.0001), BMI (p < 0.0001), and waist/hip ratio (p < 0.0001). More overweight subjects were found in the non-IR group (50% vs. 34.64%, p = 0.02), whereas the number of obesity subjects was higher in the IR group (59.05% vs. 23.12%, p < 0.0001). Out of overall study population, 45 subjects had atherogenic dyslipidemia (28.35% in the IR group, and 8.03% in the non-IR group; p < 0.0001), 110 had MetS (67.71% in the IR group and 21.42% in the non-IR group; p < 0.0001), whereas 136 of them were hypertensive (71.31% in the IR group and 43.75% in the non-IR group, p < 0.0001).

**Table 1:** Characteristics of the study subjects, as stratified by insulin resistance status

Variable	Insulin resistance	No insulin resistance	p
N (F)	122 (7)	112 (12)	
Age; years	41.00 (39.62, 42.38)	40.00 (38.52, 41.47)	0.39
Metabolic syndrome; n (%)	86 (67.71)	24 (21.42%)	< 0.0001
Atherogenic dyslipidemia; n (%)	36 (28.35)	9 (8.03)	0.0001
FRS	5.40 (4.26, 6.53)	3.20 (2.22, 4.17)	0.0001
BMI; kg/m^2^	31.44 (30.55, 32.32)	27.75 (26.95, 28.55)	< 0.0001
W/H ratio	0.977 (0.973, 0.990)	0.91 (0.895, 0.926)	< 0.0001
Overweight; n (%)	44 (34.64)	58 (51.78)	0.01
Obesity; n (%)	78 (61.42)	25 (23.21)	< 0.0001
Stable CHD, n (%)	13 (10.23%)	4 (3.57%)	0.06
Coronary plaque; n (%)^a^	32 (33.33)	13 (21.31)	0.11
Hypertension; n(%)	87 (71.31)	49 (43.75)	< 0.0001
Current smoking; n (%)	42 (33.07)	35 (31.25)	0.98
Total cholesterol; mmol/L	5.40 (5.18, 5.61)	5.05 (4.81, 5.28)	0.08
LDL cholesterol; mmol/L	3.12 (2.94, 3.30)	2.99 (2.80, 3.17)	0.32
HDL cholesterol; mmol/L	1.20 (1.15, 1.24)	1.29 (1.25, 1.34)	0.01
Triglycerides, mmol/L	2.05 (1.68, 2.42)	1.34 (1.05, 1.62)	< 0.001
Glucose; mmol/L	5.40 (5.24, 5.57)	5.10 (4.99, 5.20)	< 0.0001
ALT; U/L	44.00 (40,49, 49.50)	38.00 (32,82, 39.77)	0.002
AST; U/L	34.00 (31.89, 36.90)	30.00 (28.07, 31.37)	0.002
Total bilirubin; µmol/L	12.00 (10.42, 13.57)	13.00 (11.53, 14.46)	0.16
Uric acid; µmol/L	378.00 (359.61, 396.38)	334.50 (315.95, 353.04)	< 0.0001
Serum creatinine; µmol/L	91.00 (87.78, 94.21)	89.00 (85.84, 92.15)	0.30
SBP; mm Hg	135.00 (131.40, 138.66)	120.00 (115.92, 124.07)	< 0.0001
DBP; mm Hg	85.00 (82.65, 87.35)	80.00 (76.56, 82.43)	0.0009
cIMT; mm	0.56 (0.550, 0.571)	0.54 (0.535, 0.547)	0.001
FMD; %	7.08 (6.30, 7.85)	7.57 (6.75, 8.38)	0.31
CRP; mg/L	1.29 (1.229, 1.36)	0.88 (0.53, 1.23)	0.0001
TNF-α; pg/mL	0.92 (0.90, 0.94)	0.82 (0.74, 0.89)	0.02
Fibrinogen; g/L	3.30 (3.11, 3.48)	3.20 (3.01, 3.38)	0.61
Insulin; µIU/mL	23.82 (20.10, 27.54)	11.37 (10.55, 12.18)	< 0.0001
HOMA-IR	3.02 (2.78, 3.26)	1.46 (1.35, 1.56)	< 0.0001
Plasma 8-iso-PGF_2α_; pg/mL	7.60 (6.55, 8.03)	6.39 (5.21, 6,56)	0.04
Urine cortisol; ng/mg of creatinine	119.70 (105.01, 134.40)	89.80 (76.90, 102.69)	< 0.001
PSS-10 score	17.00 (15.73, 18.27)	16.0 (14.86, 17.30)	0.62
Medications, n (%)			
Aspirin	1 (0.82)	5 (4.46)	0.07
ACEI/ARB	31 (25.40)	10 (8.93)	0.001
β-blocker	28 (22.95)	12 (10.71)	0.01
Ca-blocker	10 (8.10)	3 (2.68)	0.05
Diuretics	22 (18.03)	5 (4.46)	0.001
Lipid-lowering agents	7 (5.74)	4 (3.57)	0.43

Data are expressed as no. (%), or median (95% CI)

Framingham Risk Score, 10-year risk of developing coronary heart disease; *CHD* coronary heart disease, *BMI* body mass index, *W/H* waist/hip, *FMD* flow-mediated dilation, *cIMT* carotid intima-media thickness, *LDL* low density lipoprotein, *HDL* high density lipoprotein, *CRP* C-reactive protein, *8-iso-PGF*_*2α*_ 8-iso-prostaglandin F_2α_, *TNF-α* tissue necrotic factor-α, *SBP (DBP)* systolic (diastolic) blood pressure, *HOMA-IR* the homeostasis model assessment of insulin resistance, *PSS* perceived stress scale, *ALT* alanine aminotransferase, *AST* aspartate aminotransferase

^a^n (%) subjects who underwent computed tomography coronary angiography

In the non-IR subjects, only in 20 (9.09%) of them HOMA-IR value was ≤ 1.0. Also, the respective groups (IR *vs.* non-IR) significantly differed in the variables whose values, when exceeding the reference range (i.e. abnormal), are acknowledged the key identification attributes of MetS (i.e., HDL cholesterol, triglycerides, glucose, blood pressure, and waist circumference (not comprised in the table). Furthermore, the levels of pro-inflammatory markers (CRP, TNF-α) were also significantly higher in the IR subjects, as compared to the non-IR ones (Table 1).

The median (95% CI) level of perceived stress was 17.00 (15.73, 18.27), and 16.0 (14.86, 17.30), (p = n.s.) for the IR and non-IR subjects, respectively. Urine median (95% CI) cortisol (calculated as ng/mg of creatinine) was significantly higher in the IR subjects [119.70 (105.01, 134.40) vs. 89.80 (76.90, 102.69), p < 0.001]. The IR subjects also had significantly higher plasma median (95% CI) 8-iso-PGF_2α_ level, as compared to the non-IR ones [7.60 (6.55, 8.03) pg/mL vs. 6.39 (5.21, 6,56) pg/mL, p = 0.04]. The results of univariate analyses regarding the associations between the indices of psychological stress (PSS, cortisol) and oxidative stress marker are presented in Table 2. A positive association between the perceived stress level and cortisol was established [B = 2.150, 95% CI (0.212, 4.097), p = 0.03], and both psychological stress indices were positively associated with plasma 8-iso-PGF_2α_ [B = 0.139, 95% CI (0.048, 0.230), p = 0.002, and B = 0.007, 95% CI (0.0006, 0.014), p = 0.03; for PSS score and cortisol, respectively].

**Table 2:** Associations between the indices of psychological stress and oxidative stress marker in univariate analyses

	Cortisol		PSS scores	
	B (95% CI)	p	B (95% CI)	p
8-iso-PGF_2α_	0.007 (0.0006, 0.014)	0.03	0.139 (0.048, 0.230)	0.002
Cortisol	–	–	2.150 (0.202, 4.097)	0.03

*B* unstandardized coefficient, *CI* confidence interval, *8-iso-PGF*_*2α*_ 8-iso-prostaglandin F_2α_

As evidenced in the univariate analysis, HOMA-IR value was associated with plasma 8-iso-PGF_2α_ level [B = 0.069, 95% CI (0.016, 0.120), p = 0.01], whereas with respect to both psychological stress indices, such an association was confirmed for cortisol only [B = 0.003, 95% CI (0.0003, 0.005), p = 0.02] (Table 3). In the multivariate models (comprising plasma 8-iso-PGF_2α_, urine cortisol, PSS score), adjusted for age, sex, and current smoking, 8-iso-PGF_2α_ was associated with HOMA-IR [B = 0.068; 95% CI (0.007, 0.129), p = 0.02], whereas the association between cortisol and HOMA-IR approximated the significance level (B = 0.002, 95% CI [-0.0003, 0.005], p = 0.06; Table 3). No associations were established between PSS score and HOMA-IR (B = 0.007, 95% Cl [-0.028, 0.043], p = 0.67 and B = -0.013, 95% CI [-0.050, 0.023], p = 0.48), neither in univariate, nor multivariate analyses.

**Table 3:** Associations between the indices of psychological stress and oxidative stress marker with HOMA-IR

	B (95% CI)	p
8-iso-PGF_2α_	0.069 (0.016, 0.120)	0.01
	0.068 (0.007, 0.129)	0.02^#^
Cortisol	0.003 (0.0003, 0.005)	0.02
	0.002 (− 0.0003, 0.005)	0.06^#^
PSS scores	0.007 (− 0.028, 0.043)	0.67
	− 0.013 (− 0.050, 0.023)	0.48^#^

*B* unstandardized coefficient, *CI* confidence interval, *HOMA-IR* the homeostasis model assessment of IR, *8-iso-PGF*_*2α*_ 8-iso-prostaglandin F_2α_

^#^Multivariate regression model comprised cortisol, PSS score, 8-iso-PGF_2α_, adjusted for age, sex, and current smoking

As evidenced in Table 4, the associations of 8-iso-PGF_2α_ (B = 0.032, 95% CI [-0.013, 0.078], p = 0.11) and cortisol (B = 0.001, 95% CI [-0.0007, 0.003], p = 0.18) with HOMA-IR became non-significant. Out of the variables addressed in the regression model, waist circumference (B = 0.056, 95% CI [0.039, 0.074], p < 0.0001) and systolic blood pressure (B = 0.009, 95% CI [0.00003, 0.018], p = 0.04) were positively associated with HOMA-IR, whereas the association of HDL cholesterol (B = -0.597, 95% CI [-1.139, -0.055], p = 0.03) was a negative one (Table 4).

**Table 4:** Associations of select variables with HOMA-IR in multivariate analysis

Variable	B (95% CI)	p
Waist circumference	0.056 (0.039, 0.074)	< 0.0001
Triglycerides	− 0.531 (− 0.174, 0.069)	0.39
HDL cholesterol	− 0.597 (− 1.139, − 0.055)	0.03
SBP	0.009 (0.00003, 0.018)	0.04
8-iso-PGF_2α_	0.032 (− 0.013, 0.078)	0.11
Cortisol	0.001 (− 0.0007, 0.003)	0.18

Multivariate regression model comprised of the variables whose values, when exceeding the reference range (i.e. abnormal), are the key identification attributes of metabolic syndrome (apart from glucose), cortisol, and 8-iso-PGF_2α_, adjusted for age, sex, and current smoking

*B* unstandardized coefficient, *CI* confidence interval, *SBP* systolic blood pressure, *8-iso-PGF*_*2α*_ 8-iso-prostaglandin F_2α_

Crude and adjusted OR regarding the impact of cortisol, 8-iso-PGF_2α_, atherogenic dyslipidemia and MetS (per se), as well as the MetS components, on the incidence of IR, are presented in Tables 5 and 6. In the univariate model, cortisol (OR = 1.007, 95% CI [1.002, 1.012], p = 0.006), and 8-iso-PGF_2α_ (OR = 1.103, 95% CI [1.010, 1.201], p = 0.02) affected the incidence of IR. Even though the effect of plasma 8-iso-PGF_2α_ was small, it nevertheless increased the chances for the incidence of IR by 10%. MetS (as a binary variable) had the strongest impact on the incidence of IR (OR = 6.828, 95% CI [3.775, 12.354], p < 0.00001), while atherogenic dyslipidemia (as a binary variable) also proved significant (OR = 3.511, 95% CI [1.634, 7.553], p = 0.001) (Table 5). In the multivariate logistic regression model, adjusted for age, sex, current smoking, and atherogenic dyslipidemia, the impact of cortisol [OR = 1.006, 95% CI (1.001, 1.011), p = 0.01] and 8-iso-PGF_2α_ [OR = 1.011, 95% CI (1.002, 1.213), p = 0.04] on the incidence IR was still significant. On the other hand, after adjustment for MetS, instead of atherogenic dyslipidemia, age, sex, and current smoking as the correcting variables, the effects of 8-iso-PGF_2α_ [OR = 1.017, 95% Cl (0.922, 1.120), p = 0.72] and cortisol [OR = 1.004, 95% CI (0.999, 1.010), p = 0.08] became attenuated, and consequently non-significant (Table 5).

**Table 5:** Odds ratios for the incidence of insulin resistance

Variable	OR (95% CI)	p
8-iso-PGF_2α_	1.103 (1.010, 1.203)	0.02
	1.011 (1.002, 1.213)	0.04^#^
	1.017 (0.922, 1.120)	0.72^¶^
Cortisol	1.007 (1.002, 1.012)	0.006
	1.006 (1.001, 1.011)	0.01^#^
	1.004 (0.999, 1.010)	0.08^¶^
Atherogenic dyslipidemia	3.511 (1.634, 7.553)	0.001
Metabolic syndrome	6.828 (3.775, 12.354)	< 0.00001

*OR* odds ratio, *CI* confidence interval, *8-iso-PGF*_*2α*_ 8-iso-prostaglandin F_2α_

^#^Multivariate regression model comprised 8-iso-PGF_2α_, cortisol, atherogenic dyslipidemia (yes/no), adjusted for age, sex, and current smoking

^¶^Multivariate regression model comprised 8-iso-PGF_2α_, cortisol, metabolic syndrome (yes/no), adjusted for age, sex, and current smoking

**Table 6:** Odds ratios for insulin resistance relative to metabolic syndrome components, cortisol, and 8-iso-PGF_2α_

Variable	OR (95% CI)	p
Waist circumference^a^	4.966 (2.29, 10.75)	0.00004
	3.621 (1.602, 8.182)	0.001^#^
High blood pressure	1.860 (0.976, 3.542)	0.05
	1.940 (0.966, 3.896)	0.06^#^
Hypertriglyceridemia	1.993 (1.063, 3.736)	0.03
	2.012 (1.025, 3.949)	0.04^#^
Low HDL cholesterol	1.472 (0.706, 3.068)	0.29
	1.568 (0.726, 3.386)	0.24^#^
8-iso-PGF_2α_	1.055 (0.953, 1.168)	0.29^#^
Cortisol	1.005 (0.999, 1.100)	0.10^#^

*OR* odds ratio, *CI* confidence interval, *8-iso-PGF*_*2α*_ 8-iso-prostaglandin F_2α_

^#^Baseline model, cortisol, and 8-iso-PGF_2α_ adjusted for age, sex, and current smoking

^a^≥94/80 cm (men/women). Baseline model comprised of metabolic syndrome components, except elevated fasting plasma glucose, as glucose was already included in the HOMA-IR formula

In turn, in the multivariate logistic regression model, taking into account the MetS components, cortisol, and 8-iso-PGF_2α_, only waist circumference [OR 4.966, 95% CI (2.29, 10.751), p = 0.00004] and hypertriglyceridemia [OR 1.993, 95% CI (1.063, 3.736), p = 0.03] affected the incidence of IR, while the effect of high blood pressure approached the significance level [OR 1.860, 95% CI (0.976, 3.542), p = 0.05] (Table 6). Following additional adjustment for age, sex, and current smoking, these associations persisted.

In the univariate models, it was established that plasma 8-iso-PGF_2α_ level was positively associated with systolic blood pressure [B = 0.032, 95% Cl (0.008, 0.055), p = 0.008], waist circumference [B = 0.047, 95% Cl (0.011, 0.083), p = 0.01], and CRP level [B = 0.257, 95% Cl (0.014, 0.502), p = 0.03]. Positive associations between both inflammatory markers and waist circumference [B = 0.053, 95% Cl (0.034, 0.071) p < 0.0001, and B = 0.026, 95% Cl (0.0005, 0.052) p = 0.04; for CRP and TNF-α, respectively] were also corroborated (data not comprised in the table).

No correlation was established between the level of perceived stress and inflammatory markers (ρ = 0.02, p = 0.80, and ρ = 0.02, p = 0.75, for CRP and TNF-α, respectively), while urine cortisol positively correlated with CRP only (ρ = 0.21, p = 0.001). Both inflammatory markers positively correlated with HOMA-IR value (ρ = 0.26, p < 0.0001, and ρ = 0.16, p = 0.01, for CRP and TNF-α, respectively). Neither were there any correlations established between the actual duration of police work and PSS score (ρ = 0.002, p = 0.97), urine cortisol (ρ = -0.07, p = 0.25), plasma 8-iso-PGF_2α_ level (ρ = 0.009, p = 0.88), and HOMA-IR (ρ = -0.09, p = 0.15) value (data not comprised in the table).

## Discussion

In the present cross-sectional study, positive associations between the indices of psychological stress, i.e. self-reported perceived stress, and cortisol (biological stress marker) with plasma 8-iso-PGF_2α_ were established. We also demonstrated the associations of cortisol and plasma oxidative stress marker with HOMA-IR and the incidence of IR in the subjects exposed to a highly stressful occupation.

Studies on rodents and humans give grounds to the belief that oxidative stress is an important trigger for IR, while research in humans regarding the association between psychological stress and oxidative stress is generally scarce [13, 15, 20–22]. Perceived stress in the pre-menopausal caregiving women was not related to urine isoprostanes, but to the oxidative stress index [20]. Also, in the study of Shimanoe et al. the association between perceived stress and the excretion of 8-hydroxydeoxyguanosine (oxidative damage DNA marker) in urine was established as a weak one [22].

Even though no significant difference in perceived stress between respective groups was established, the mean values of PSS score in IR and non-IR subjects were higher than in the other populations under study, whilst remaining in full compliance with the norms proposed by Cohen [23, 24]. The mean score of perceived stress in our IR subjects was also higher (17.08; 42.70%) than in the Lesage et al. study of population aged 41-50 years (16.1; 40.25%) [24]. In the two studies on police officers perceived stress values (as assessed by PSS-10, or PSS-14 scale) were lower than in our own study. In a Chinese study, only women participated, as well as were younger than our population, while in the Midwestern police force officers study, only about 61% of the study population were active duty policemen [25, 26].

A positive association between the scores of perceived stress and urine cortisol levels in our population was also demonstrated in other studies focused on police officers [27, 28]. On the other hand, Mikkelsen et al. in a large cohort study of Danish public service employees, found neither any cross-sectional, nor any longitudinal impact of prolonged perceived stress on the level on morning salivary cortisol [29]. Plasma, as well as salivary cortisol levels were found higher prior to an anticipatory emotional/cognitive threat, as well as in response to acute mental stress in firefighters and police officers, as compared to the controls [30–32].

Positive relationships between the self-reported perceived stress and cortisol with plasma 8-iso-PGF_2α_, as well as the association between both psychological stress indices seem to point to a possible link between the activation HPA response to psychological stressors and metabolic cortisol effects. We construe the results yielded by the present study in the light of the hypothesis proposed by Aschbacher et al. whereby “heightened anticipatory cortisol reactivity would mediate the relationship between perceived stress and elevated oxidative stress damage”. It was documented that heightened anticipatory salivary cortisol responses to psychological stress were associated with the higher levels of plasma 8-iso-PGF_2α_ [33].

Besides, the urine samples had been taken before their shift, higher urine cortisol levels in the IR subjects may indicate greater anticipatory cortisol response, and may therefore be well indicative of the links between the perceived psychological stress and an increased susceptibility to oxidative stress, e.g. increased plasma 8-iso-PGF_2a_ may appear appreciably later than the adrenal cortisol release. Furthermore, it was suggested that urinary free cortisol excretion at the night-time period and early morning hours were most likely an optimal time frame for investigating the subtle alterations of the HPA axis activity in obese individuals [34]. Response to stressors and their effect on cortisol secretion may, to some extent, be attributable to both the actual type, and perceived intensity of the stressor by an individual officer, i.e. a “stimulus response specificity”, and that events or conditions considered highly stressful by the police officers may be associated with the disturbances of a typical awakening cortisol pattern [35, 36].

In contrast to our cross-sectional study of non-diabetic population, self-perceived stress was an independent predictor of incident type 2 diabetes in long term follow-up study of Novak et al. [4]. Also, in yet another recently published study, perceived stress emerged as a significant predictor of type 2 diabetes mellitus, even though the association of PSS score with HOMA-IR was not assessed [7]. On the other hand, subclinical hypercortisolism in elderly patients with type 2 diabetes, as well as higher urinary free cortisol in overweight girls, associated with visceral adiposity and IR, was reported [37, 38].

Joseph et al. demonstrated, that cortisol curve features were, paradoxically, associated with lower HOMA-IR in the non-diabetic participants [38, 39]. In the normoglycemic subjects, cortisol inhibits insulin release, while chronic psychological stress may impair the feedback mechanisms which return these hormonal systems to normal, resulting in chronic elevation in cortisol levels [9]. Our subjects with weight gain/body fat may lose the beneficial effect of the interaction between cortisol and the adipoinsular axis, which consequently accounts for the detrimental effects of cortisol, including lipolysis, IR, and other metabolic effects [39]. This hypothesis is supported by Darmon et al. in whose study a low-dose hydrocortisone infusion, administered to simulate mild stress, developed markedly greater IR in abdominally obese participants, as compared to normal BMI controls [40]. Measurement of single-point urine cortisol only may be less sensitive in terms of detecting subtle differences in subclinical hypercortisolism, and not actually reflect the total cortisol load. We did nevertheless manage to demonstrate its association with oxidative stress and HOMA-IR.

The oxidative stress markers were found higher in the animal study [41]. Data pertaining to the relationship between oxidative stress and IR in humans are scarce, and none are specifically focused on police officers [15, 17, 21]. In the present study, plasma 8-iso-PGF_2α_ was positively related to HOMA-IR, and increased by 10% the chance of IR incidence in a middle-aged population burdened with psychological stress. Atherogenic dyslipidemia (higher triglycerides and low HDL cholesterol) often precedes the full clinical manifestation of MetS, and is associated with IR. When adjusting for it, however, the impact of oxidative stress marker on IR incidence was still evident. MetS, and waist circumference (originating in its components), most manifestly affected the development of IR, as corroborated by other investigators [15–17]. After adjustment for BMI, or waist circumference, no relationships plasma F2-isoprostanes with HOMA-IR were reported in longitudinal Park et al. study, while in the other a cross-sectional study, relationship of urine 8-iso-PGF_2α_ was established as BMI-independent with regard to HOMA-IR only, although not with regard to IR [15, 21].

In the highly developed countries, IR occurs in 24–50% of adults, and remains consistently on the rise. To the best of our knowledge, the present study is the first one ever to highlight the interrelationships between oxidative stress and IR, which may well be regarded as the metabolic consequences of psychological stress in police officers. In contrast to physiological conditions, in a stressful situation, anabolic-catabolic imbalance (high cortisol, insulin) and low concentration of the opposite hormones, may lead to oxidative stress, systemic inflammation, and IR. Excessive caloric intake and low energy expenditure increase ROS production in the mitochondria [42]. It is also well-worth noting that ingestion of calories-rich foods during stressful periods contributes to obesity, which also plays a key role in the development of IR.

The exact pathomechanism of oxidative stress being instrumental in IR development has not yet been fully elucidated, although acknowledged to entail a disruption of complex network of insulin signalling pathways. Metabolic disturbances (i.e. enhanced plasma fatty acids level, hyperglycaemia and hyperinsulinemia) in peripheral IR also promote of IR brain and neurotoxicity, which may manifest themselves through the disturbances in neurotransmitter’s release and uptake [43]. ROS also stimulate a pro-inflammatory signalling pathway, linking oxidative stress with an inflammatory state and IR. Both inflammatory markers (CRP, TNF-α) were positively associated with waist circumference (obesity indicator) in the study subjects. An enhanced production of TNF-α by adipocytes induce liver CRP synthesis in obese subjects, indicating an activation of a pro-inflammatory signalling pathway, which may have contributed to the development of IR.

The correlations between plasma inflammatory markers and HOMA-IR in present study were also documented. Positive association between 8-iso-PGF_2α_ and CRP, as established throughout our study, remains well in line with the findings of Black et al. [44]. This is an important observation, as CRP is involved in the progression of atherosclerosis at every stage of its development, as well as contributes to CVD risk. The bidirectional association between oxidative stress and inflammation is also demonstrated by other investigators [45, 46]. On the other hand, reduction of ROS production enhances both mitochondrial function, and insulin sensitivity [47].

Our study revealed frequent incidence of metabolic CVD risk factors, and the links of psychological and oxidative stress with IR in police officers. It follows that implementation of an integrated prevention strategy, based on a systematic evaluation of the total risk of a disease at an individual level, appears essential for this particular occupational group. The subjects at risk would then be identified much earlier in the course of a vascular disease, while the attendant risk factors would stand a good chance of being mitigated, while clinical manifestations of CVD, as well as other consequences of obesity and MetS might also be appreciably reduced through adequate control regimen.

### Limitations

Several likely limitations of the present study need to be acknowledged. Firstly, we are aware that the methods used to assess psychological stress in our study may not reflect the actual level of stress in the population sample. We assessed the subjective stress perception, even though we were unable to evaluate any specific sources of stress in the actual police work, for reasons beyond our control. Secondly, we addressed one marker of oxidative stress only, without pursuing assessment of antioxidative enzymes and other antioxidative substance which would provide information about the redox homeostasis in vivo. Thirdly, in our cohort of police officers, only a single-point urine cortisol measurement was taken.

Admittedly, measuring the trajectory of morning salivary cortisol (or diurnal cortisol secretion), might well yield more information on total cortisol load, as well as on the actual nature of the relationships under study. Fourthly, the cut-off HOMA-IR value in the present study was 2.04 (see the Methods section), based on the receiver operating characteristics (ROC) analysis. In other studies, various HOMA-IR cut-off values were used to determine IR, e.g. in the study by Gayoso-Diz et al. the cut-off values were 1.60 vs. 2.05 for diabetic and non-diabetic men with MetS, respectively (defined in line with IDF criteria) [48]. Furthermore, the study subjects differed in their CVD risk factors and medication intake which might also affect the results. The results of our own study may not stand an effective comparison with other studies for several reasons, e.g. substantial differences in the populations under study, age, and other concomitant metabolic disorders. Finally, as the present study is of a cross-sectional design, the actual causal relationships do not really lend themselves to accurate inferences.

## Conclusions

To the best of our knowledge, the present study is the first one to highlight the interrelationships between oxidative stress and IR, which may well be regarded as the metabolic consequences of psychological stress, as psychological stress was associated with an oxidative one. In the subjects exposed to frequent/chronic psychological stressors, cortisol, and oxidative stress marker were associated with IR, being statistically attenuated, though, following adjustment for MetS or its components. Further studies are nevertheless required to have these findings effectively corroborated, also with a view to addressing any potential relationships between different variables.

## Data Availability

The datasets generated during and/or analysed during the current study are not publicly available due to statutory confidentiality constraints applicable to police work, but are available in a restricted scope from the corresponding author on reasonable request.

## References

[1] Patel TP, Rawal K, Bagchi AK, Akolkar G, Bernardes N, Dias DDS (2016). Insulin resistance: an additional risk factor in the pathogenesis of cardiovascular disease in type 2 diabetes. Heart Fail Rev.

[2] Gami AS, Witt BJ, Howard DE, Erwin PJ, Gami LA, Somers VK, Montori VM (2007). Metabolic syndrome and risk of incident cardiovascular events and death. A systematic review and meta-analysis of longitudinal studies. J Am Coll Cardiol..

[3] McEwen BS (1998). Protective and damaging effects of stress mediators. N Engl J Med.

[4] Novak M, Bjorck L, Giang KW, Heden-Stahl C, Wilhelmsen L, Rosengren A (2013). Perceived stress and incidence of Type 2 diabetes: a 35-year follow-up study of middle-aged Swedish men. Diabet Med..

[5] Janczura M, Bochenek G, Nowobilski R, Dropinski J, Kotula-Horowitz K, Laskowicz B (2015). The relationship of metabolic syndrome with stress, coronary heart disease and pulmonary function—an occupational cohort-based study. PLoS ONE.

[6] Cohen S, Kamarck T, Mermelstein RA (1983). A global measure of perceived stress. J Health Soc Behav.

[7] Madhu SV, Siddiqui A, Desai NG, Sharma SB, Bansal AK (2019). Chronic stress, sense of coherence and risk of type 2 diabetes mellitus. Diabetes Metab Syndr..

[8] Kahn SE, Hull RL, Utzschneider KM (2006). Mechanisms linking obesity to insulin resistance and type 2 diabetes. Nature.

[9] Joseph JJ, Golden SH (2017). Cortisol dysregulation: the bidirectional link between stress, depression, and type 2 diabetes mellitus. Ann N Y Acad Sci.

[10] Teague CR, Dhabhar FS, Barton RH, Beckwith-Hall B, Powell J, Cobain M (2007). Metabonomics studies on the physiological effects of acute and chronic psychological stress in Sprague-Dawley rats. J Proteome Res.

[11] Spiers JG, Chen HJ, Sernia C, Lavidis NA (2015). Activation of the hypothalamic-pituitary-adrenal stress axis induces cellular oxidative stress. Front Neurosci..

[12] Keaney JF, Larson MG, Vasan RS, Wilson PW, Lipinska I, Corey D (2003). Obesity and systemic oxidative stress: clinical correlates of oxidative stress in the Framingham Study. Arterioscler Thromb Vasc Biol.

[13] Houstis N, Rosen ED, Lander ES (2006). Reactive oxygen species have a causal role in multiple forms of insulin resistance. Nature.

[14] Morrow JD, Hill KE, Burk RF, Nammour TM, Badr KF, Roberts LJ (1990). A series of prostaglandin F2-like compounds are produced in vivo in humans by a non-cyclooxygenase, free radical-catalyzed mechanism. Proc Natl Acad Sci USA.

[15] Meigs JB, Larson MG, Fox CS, Keaney JF, Vasan RS, Benjamin EJ (2007). Association of oxidative stress, insulin resistance, and diabetes risk phenotypes. The Framingham Offspring Study. Diabetes Care..

[16] Costabile G, Della Pepa G, Bozzetto L, Annuzzi G, Vetrani C, Giacco R (2015). Urine 8-isoprostane in relation to adiposity and insulin resistance in individuals at high cardiometabolic risk. Metab Syndr Relat Disord..

[17] D’Archivio M, Annuzzi G, Vari R, Filesi C, Giacco R, Scazzocchio B (2012). Predominant role of obesity/insulin resistance in oxidative stress development. Eur J Clin Invest.

[18] Alberti KG, Ecel RH, Grundyk SM, Zimmet PZ, Cleeman JI, Donato KA (2009). Harmonizing the metabolic syndrome: a joint interim statement of the International Diabetes Federation Task Force on Epidemiology and Prevention; National Heart, Lung, and Blood Institute; American Heart Association; World Heart Federation; International Atherosclerosis Society; and International Association for the Study of Obesity. Circulation.

[19] Morrow JD, Roberts LJ (1999). Mass spectrometric quantification of F2-isoprostanes in biological fluids and tissues as measure of oxidant stress. Methods Enzymol..

[20] Epel ES, Blackburn EH, Lin J, Dhabhar FS, Adler NE, Morrow JD, Cawthon RM (2004). Accelerated telomere shortening in response to life stress. Proc Natl Acad Sci USA..

[21] Park K, Gross M, Lee DH, Holvoet P, Himes JH, Shikany JM, Jacobs DR (2009). Oxidative stress and insulin resistance: the coronary artery risk development in young adults. Diabetes Care.

[22] Shimanoe C, Hara M, Nishida Y, Nanri H, Horita M, Yamada Y (2018). Perceived stress, depressive symptoms, and oxidative DNA damage. Psycosom Med..

[23] Cohen S, Williamson G, Spacapan S, Oskamp S (1988). Perceived stress in a probability sample of the United States. The social psychology of health: claremont symposium on applied social psychology.

[24] Lesage F, Berjot S, Deschamps F (2012). Psychometric properties of the French versions of the perceived stress scale. Int J Occup Med.

[25] Wang Z, Chen J, Boyd JE, Zhang H, Jia X, Qiu J, Xiao Z (2011). Psychometric properties of the chinese version of the perceived stress scale in policewomen. PLoS ONE.

[26] Ramey SL, Perkhounkova Y, Downing NR, Culp KR (2011). Relationship of cardiovascular disease to stress and vital exhaustion in an urban, midwestern police department. AAOHN J..

[27] van Eck M, Nicolson N (1994). Perceived stress and salivary cortisol in daily life. Ann Behav Med.

[28] Walvekar SS, Ambekar JG, Devaranavadagi BB (2015). Study on serum cortisol perceived stress scale in the police constables. J Clin Diag Res..

[29] Mikkelsen S, Forman JL, Fink S, Vammen MA, Thomsen JF, Grynderup MB (2017). Prolonged perceived stress and saliva cortisol in a large cohort of Danish public service employees: cross-sectional and longitudinal associations. Int Arch Occup Environ Health.

[30] Rosati MV, Sancini A, Tomei F, Andreozzi G, Scimitto L, Schifano MP (2011). Plasma cortisol concentrations and lifestyle in a population of outdoor workers. Int J Environ Health Res..

[31] Robinson SJ, Leach J, Owen-Lynch PJ, Sünram-Lea SI (2013). Stress reactivity and cognitive performance in a simulated firefighting emergency. Aviat Space Environ Med.

[32] Groer M, Murphy R, Bunnell W, Salomon K, Van Eepoel J, Rankin B, White K, Bykowski C (2010). Salivary measures of stress and immunity in police officers engaged in simulated critical incident scenarios. J Occup Environ Med.

[33] Aschbacher K, O’Donovan A, Wolkowitz OM, Dhabhar FS, Su Y, Epel E (2013). Good stress, bad stress and oxidative stress: insights from anticipatory cortisol reactivity. Psychoneuroendocrinology..

[34] Pasquali R (2012). The hypothalamic–pituitary–adrenal axis and sex hormones in chronic stress and obesity: pathophysiological and clinical aspects. Ann N Y Acad Sci.

[35] Skoluda N, Strahler J, Schlotz W, Niederberger L, Marques S, Fischer S (2015). Intra-individual psychological and physiological responses to acute laboratory stressors of different intensity. Psychoneuroendocrinology..

[36] Violanti JM, Fekedulegn D, Andrew ME, Hartley TA, Charles LE, Miller DB, Burchfiel CM (2017). The impact of perceived intensity and frequency of police work occupational stressors on the cortisol awakening response (CAR): findings from the BCOPS study. Psychoneuroendocrinology..

[37] Chiodini I, Torlontano M, Scillitani A, Arosio M, Bacci S, Di Lembo S (2005). Association of subclinical hypercortisolism with type 2 diabetes mellitus: a case-control study in hospitalized patients. Eur J Endocrinol..

[38] Misra M, Bredella MA, Tsai P, Mendes N, Miller KK, Klibanski A (2008). Lower growth hormone and higher cortisol are associated with greater visceral adiposity, intramyocellular lipids, and insulin resistance in overweight girls. Am J Physiol Endocrinol Metab.

[39] Joseph JJ, Wang X, Spanakis E, Seeman T, Wand G, Needham B, Golden SH (2015). Diurnal salivary cortisol, glycemia and insulin resistance: the multi-ethnic study of atherosclerosis. Psychoneuroendocrinology..

[40] Darmon P, Dadoun F, Boullu-Ciocca S, Grino M, Alessi MC, Dutour A (2006). Insulin resistance induced by hydrocortisone is increased in patients with abdominal obesity. AJP Endocrinol Metab..

[41] Zafir A, Banu N (2009). Modulation of in vivo oxidative status by exogenous corticosterone and restraint stress in rats. Stress..

[42] Romeo GR, Lee J, Shoelson SE (2012). Metabolic syndrome, insulin resistance, and roles of inflammation–mechanisms and therapeutic targets. Arterioscler Thromb Vasc Biol.

[43] Maciejczyk M, Zebrowska E, Chabowski A (2019). Insulin Resistance and Oxidative Stress in the Brain: what’s New. Int J Mol Sci.

[44] Black CN, Bot M, Révész D, Scheffer PG, Penninx B (2017). The association between three major physiological stress systems and oxidative DNA and lipid damage. Psychoneuroendocrinology..

[45] Noren Hooten N, Ejiogu N, Zonderman AB, Evans MK (2012). Association of oxidative DNA damage and C-reactive protein in women at risk for cardiovascular disease. Arterioscler Thromb Vasc Biol.

[46] Salzano S, Checconi P, Hanschmann E, Lillig C, Bowler L, Chan P (2014). Linkage of inflammation and oxidative stress via release of glutathionylated peroxiredoxin-2, which acts as a danger signal. Proc Natl Acad Sci USA.

[47] Blendea MC, Jacobs D, Stump CS, McFarlane SI, Ogrin C, Bahtyiar G (2005). Abrogation of oxidative stress improves insulin sensitivity in the Ren-2 rat model of tissue angiotensin II over expression. Am J Physiol Endocrinol Metab.

[48] Gayoso-Diz P, Otero-González A, Rodriguez-Alvarez MX, Gude F, García F, De Francisco A, Quintela AG (2013). Insulin resistance (HOMA-IR) cut-off values and the metabolic syndrome in a general adult population: effect of gender and age: EPIRCE cross-sectional study. BMC Endocr Disord..

